# P212: Antimicrobial resistance and healthcare associated infections: one and only battle

**DOI:** 10.1186/2047-2994-2-S1-P212

**Published:** 2013-06-20

**Authors:** ML Moro, C Gagliotti, M Marchi, R Buttazzi, V Cappelli, M Morandi, F Morsillo, A Pan, M Parenti, E Ricchizzi

**Affiliations:** 1 Area di Programma Rischio Infettivo, Agenzia Sanitaria e Sociale Regione Emilia-Romagna, Bologna, Italy

## Introduction

Integrated surveillance of Healthcare infections (HAIs), antimicrobial resistance (AMR) and antimicrobial consumption (AC) is essential. Its impact in Emilia-Romagna (4.5 million inhabitants) is described.

## Methods

The following surveillance systems exist: electronic-lab-based surveillance covering all public and private hospital; AC monitoring system, covering both hospital (HA) and community; alert system of sentinel HAIs and outbreaks, both in HA and long-term care facilities (LTCFs); surgical site infection and intensive care unit surveillance system; repeated prevalence surveys both in HA and LTCFs; regional databases linkage for selected infections (eg *Clostridium difficile*); ad hoc surveillance for high priority AMR microorganisms (ie carbapenemase-producing Klebsiella pneumoniae-CPK); monitoring of HAIs and antimicrobial stewardship programs in each LHT and of hand hygiene products consumption.

## Results

Selected results are presented below. The incidence rates of bacteremia raised from 146 in 2005 to 228 cases per 100.000 inhabitants/year in 2011 (+56%); the increase was significant for K. pneumoniae (+188%) and E.coli (+99%), due to spread of multiresistant strains. An intervention program, launched in July 2011 to fight the spread of CPK, had a positive impact on this trend. The AC significantly increased until 2009; subsequently, the trend is still increasing for hospitals (90.8 DDD/100 in hospital-days in 2011) while in the community the consumption has decreased (following educational campaigns), being still high (18.4 DDD/1.000 inhabitants-day in 2011). In 2007-2011 the coverage of the regional alert system progressively improved: in 2007 26 HAI outbreaks were notified, and 54 in 2011; 17.9% occurred in LTCFs and 82.1% in HA. Data on 59,281 non orthopaedic surgeries from 33 categories of surgical procedures have been collected by 41 hospitals (2007-2011): in HAs participating to the surveillance for at least two years, the incidence of surgical wound infections was reduced by 24% (Odds Ratio 0.76, 95%CI 0.66-0.88).

## Conclusion

An integrated surveillance system, covering both HAIs and AMR, is essential to identify critical areas, to monitor interventions and to demonstrate the success of dedicated efforts.

## Disclosure of interest

None declared

